# Effect of Transcranial Direct Current Stimulation Targeting Brain Regions Identified Through Voxel‐Based Morphometry on One‐Legged Standing Balance With Eyes Closed

**DOI:** 10.1002/brb3.71425

**Published:** 2026-04-22

**Authors:** Hirona Takahashi, Yasuto Inukai, Kazuaki Nagasaka, Shota Miyaguchi, Noriko Sakurai, Naoki Kodama, Naofumi Otsuru, Hideaki Onishi

**Affiliations:** ^1^ Graduate School Niigata University of Health and Welfare Niigata‐shi Niigata Japan; ^2^ Institute for Human Movement and Medical Sciences Niigata University of Health and Welfare Niigata‐shi Niigata Japan; ^3^ Department of Physical Therapy Niigata University of Health and Welfare Niigata‐shi Niigata Japan; ^4^ Department of Radiological Sciences Gunma Paz University Takasaki Gunma Japan; ^5^ Department of Radiological Technology Niigata University of Health and Welfare Niigata‐shi Niigata Japan

**Keywords:** balance, cerebellum, transcranial direct current stimulation, voxel‐based morphometry

## Abstract

**Introduction:**

Voxel‐based morphometry (VBM) is a neuroimaging technique for quantitative analysis of brain structure. Previous studies have reported an association between gray matter (GM) volume in specific brain regions and balance function. Anodal transcranial direct current stimulation (tDCS) enhances excitability beneath the stimulation site and facilitates motor function. However, it remains unclear which brain regions are associated with one‐legged standing (OLS) balance with eyes closed and whether anodal tDCS targeting these regions enhances this balance function. The present study aimed to (i) identify brain regions associated with OLS balance with eyes closed using VBM and (ii) test whether anodal tDCS applied to the identified region enhances this balance function.

**Methods:**

The study consisted of two experiments. In Experiment 1, magnetic resonance imaging scans were performed in 66 healthy participants who underwent measurement of OLS balance time with eyes closed. In Experiment 2, brain regions identified through VBM analysis were stimulated with anodal tDCS, and the effect on OLS balance with eyes closed was evaluated. Thirty‐two healthy participants were randomly assigned to the tDCS or sham group, and OLS duration with eyes closed was assessed before and after stimulation.

**Results:**

In Experiment 1, whole‐brain analysis revealed that the cerebellar posterior vermis was associated with OLS balance with eyes closed. In Experiment 2, OLS duration with eyes closed was significantly increased poststimulation in the tDCS group after targeting the cerebellar vermis, including posterior vermis, while no significant difference was found between pre‐ and poststimulation in the sham group.

**Conclusion:**

GM volume of the cerebellar posterior vermis is associated with OLS balance with eyes closed. Anodal tDCS targeting the cerebellar vermis, including the posterior vermis, enhances this balance function.

## Graphical abstract

Anodal tDCS targeting the cerebellar vermis, identified through VBM, improves one‐legged standing balance with eyes closed.

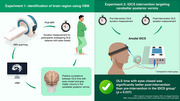

## 1 Introduction

The ability to maintain one‐legged balance is fundamental for performing a wide range of activities, from daily life to sports (Hrysomallis 2011; Vellas et al. 1997). Among these, one‐legged standing (OLS) balance with eyes closed is a method used for assessing balance function (Bohannon et al. 1984; Briggs et al. 1989; Franchignoni et al. 1998; Vereeck et al. 2008). Impaired balance during OLS with eyes closed is commonly observed in middle‐aged adults (40–60 years), and performance varies considerably among younger healthy adults (Morioka et al. 2012; Springer et al. 2007). Furthermore, it has been reported that poor balance during OLS with eyes closed is associated with an increased risk of ankle sprains and other related injuries (McGuine et al. 2000; Olivier et al. 2015). However, the factors underlying interindividual differences in balance function during OLS with eyes closed among young, healthy adults remain largely unknown.

Recent brain imaging analysis advances have improved the understanding of neural mechanisms underlying human balance control. Balance control engages multiple central nervous system regions that integrate sensory input from the vestibular, proprioceptive, and visual systems (Takakusaki 2017; Dijkstra et al. 2020). The cerebellum is widely considered critical for balance maintenance (Morton and Bastian 2004; Ango and dos Reis 2019), as also noted in the review by Surgent et al.; nonetheless, Surgent et al. emphasized that many prior studies relied on region‐of‐interest (ROI) analyses based on a priori hypotheses, which may overestimate cerebellar involvement (Surgent et al. 2019). Clinical population studies often highlight cerebellar contributions (Manor et al. 2010; Sullivan et al. 2010; Hocking et al. 2017), whereas in nonclinical (healthy) populations, in which static balance tasks are relatively easy, balance‐related neural correlates may extend beyond the cerebellum. In healthy individuals, greater balance ability or long‐term balance training (e.g., ballet dancers) has been associated with structural characteristics in noncerebellar cortical and subcortical regions, including the hippocampus, fusiform gyrus, and lingual gyrus (Hüfner et al. 2011). Therefore, identifying brain regions associated with OLS balance with eyes closed in young healthy adults requires an exploratory whole‐brain approach unconstrained by predefined regions. Voxel‐based morphometry (VBM) is well suited for this purpose because it enables a comprehensive assessment of associations between structural brain characteristics and behavioral measures across the whole brain (Mechelli et al. 2005; Ridgway et al. 2008). Nonetheless, to our knowledge, no study has applied VBM to examine brain regions associated with this specific balance task—OLS with eyes closed—in young healthy adults.

Noninvasive brain stimulation (NIBS) is a neuromodulation technique that uses electrical and/or magnetic stimulation to modulate neural activity in targeted brain regions (Kesikburun 2022). Among representative examples of NIBS is transcranial direct current stimulation (tDCS). tDCS is an NIBS that modulates neural activity directly beneath the stimulation site (Lefaucheur et al. 2017; Priori 2003). Previous studies have demonstrated that anodal tDCS applied to brain regions identified by VBM can enhance function associated with those regions. For example, a VBM study demonstrated that individuals with impaired finger sensation exhibit reduced gray matter (GM) volume in the primary somatosensory cortex (Schmidt‐Wilcke et al. 2018), and anodal tDCS applied to this region has been reported to enhance finger sensory function (Godde et al. 2020; Ragert et al. 2008). Similarly, individuals with impaired response inhibition exhibit reduced GM volume in the inferior frontal gyrus (Wiers et al. 2015), and anodal tDCS applied to this region can improve response inhibition (Jacobson et al. 2011; Sandrini et al. 2020; Stramaccia et al. 2015). Reports indicate that patients with depression often have reduced GM volume in the left dorsolateral prefrontal cortex (Chang et al. 2011; Bora et al. 2012). Furthermore, the application of tDCS has been reported to increase GM in this region, which is associated with symptom alleviation (Jog et al. 2023). These findings indicate that targeting brain regions that are both functionally and structurally involved with tDCS may enhance plasticity and potentially enhance performance. By identifying the brain regions associated with OLS balance with eyes closed through VBM, it may be possible to delineate the target areas for tDCS that support this balance task.

Therefore, we hypothesized that anodal tDCS targeting brain regions associated with OLS balance with eyes closed would enhance balance performance. To examine this hypothesis, we employed VBM to identify the brain regions associated with OLS balance with eyes closed and evaluate whether anodal tDCS applied to these regions improved balance function.

## 2 Participants and Methods

### 2.1 Experiments

To test our hypothesis, two experiments were conducted. Experiment 1 aimed to identify brain regions with structural features associated with OLS balance with eyes closed. Experiment 2 aimed to clarify the effects of anodal tDCS on brain regions associated with OLS balance with eyes closed, as identified through VBM.

### 2.2 Experiment 1

#### 2.2.1 Participants

A total of 66 healthy adults (30 males and 36 females; mean age: 21.2 ± 1.2 years) were enrolled. None of the participants had a history of neurological or musculoskeletal disorders. Additional inclusion criteria required that participants were eligible for magnetic resonance imaging (MRI) scanning (i.e., no claustrophobia). Exclusion criteria comprised metallic implants or electronic devices (e.g., pacemakers) and any severe psychiatric or other medical conditions that could affect study participation. Previous studies have employed varying sample sizes for VBM; however, these studies involving young, healthy adults have typically employed approximately 30–60 participants (Guan et al. 2019; Hartley and Harlow 2012; Nagasaka et al. 2022; Neumann et al. 2021; Onishi et al. 2023; Sherrill et al. 2018; Watanabe et al. 2022). Considering the sample sizes from previous studies, the present study employed a sample size of more than 60 participants to ensure a more robust and reliable analysis. All participants were provided with a comprehensive explanation of the experimental procedures, and written informed consent was obtained before participation. The present study was conducted in accordance with the principles outlined in the Declaration of Helsinki and was approved by the Ethics Committee of Niigata University of Health and Welfare (approval number: 19252–240308).

#### 2.2.2 MRI

MRI data were acquired using a 3T Vantage Galan scanner (Canon Medical Systems, Tochigi, Japan) equipped with a 32‐channel head coil (QD coil, 32‐channel head SPEEDER, Atlas SPEEDER head/neck). T1‐weighted images were acquired using the field echo three‐dimensional method with the following parameters: spatial resolution = 0.5 × 0.5 × 0.6 mm; slice thickness = 1.2 mm; time repetition = 5.8 ms; time echo = 2.7 ms; flip angle = 9°.

#### 2.2.3 OLS Balance Test With Eyes Closed

The participants underwent an OLS balance test with their eyes closed on the same day as their MRI scan. The OLS balance test with eyes closed involved asking a participant to maintain an OLS position with both arms crossed in front of the chest as long as possible. The duration a participant could keep the position with eyes closed was measured using a stopwatch. The upper limit of measurement time was 120 s, as in previous studies (Morioka et al. 2012). The criteria for terminating the measurement were determined based on previous studies and defined as follows: (1) any displacement or movement of the weight‐bearing foot on the floor during the trial, (2) contact between the floor and any body part other than the weight‐bearing foot, and (3) opening of the eyes during the closed‐eye measurement (Morioka et al. 2012). The OLS time with eyes closed was measured twice for each leg, and the mean of four measurements was calculated.

#### 2.2.4 Experimental Procedures

**FIGURE 1:**
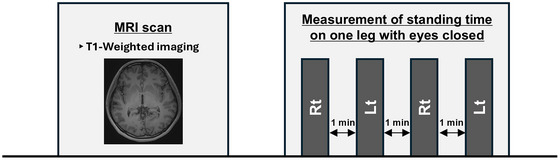
Experiment 1. All participants undergo MRI scanning to acquire T1‐weighted images, followed immediately by an OLS balance test with eyes closed. MRI, magnetic resonance imaging; OLS, one‐legged standing.

The protocol for Experiment 1 is shown in Figure 1. All participants underwent MRI scanning to acquire T1‐weighted images, followed immediately by an OLS balance test with eyes closed.

#### 2.2.5 Statistical Analysis

Image preprocessing and VBM were conducted using MATLAB R2020b (Mathworks Inc, Natick, MA, USA) and SPM12 (Statistical Parametric Mapping, Wellcome Department of Imaging Neuroscience Group, London, UK). The preprocessing was conducted in accordance with the methodology reported in our previous studies (Nagasaka et al. 2022; Onishi et al. 2023; Watanabe et al. 2022). First, the T1‐weighted images were segmented into GM, white matter, and cerebrospinal fluid regions using a unified segmentation algorithm implemented in SPM12. Spatial normalization was performed using the diffeomorphic anatomical registration through the exponentiated Lie algebra (DARTEL) algorithm. Images were then modulated (Good et al. 2001), resliced with 1.5‐mm isotropic voxels, and smoothed with an 8‐mm full width at half maximum. A multiple regression model implemented in SPM12 was used to identify brain regions associated with the OLS balance function with eyes closed. Previous studies have highlighted intracranial volume—calculated as the combined volume of gray and white matter—as a crucial covariate in VBM (Pell et al. 2008). Accordingly, intracranial volume was included as a covariate in the present study. In addition, other covariates included body mass index (BMI), which has been shown to influence OLS balance (Greve et al. 2013; Ku et al. 2012), as well as sex and handedness, which are known to affect GM volume. The dominant leg was also included to account for potential asymmetries in neuromuscular control and postural strategies, which may influence balance performance. Furthermore, handedness was assessed using the Edinburgh Handedness Inventory, and leg dominance was operationally defined as the leg preferentially used to kick a ball (Oldfield 1971; van Melick et al. 2017). Whole‐brain analysis was performed with a voxel‐level threshold of *p* < 0.005 (uncorrected) and cluster‐level threshold of *p* < 0.05, corrected for family‐wise error (FWE) (Huber et al. 2018; Wang et al. 2021). Statistical analysis was performed using a mask derived from the mean GM image across the participants.

### 2.3 Experiment 2

#### 2.3.1 Participants

A total of 32 healthy participants (19 males, 13 females; mean age: 21.0 ± 1.6 years) were randomly assigned to one of two groups: the tDCS group (*n* = 16; 10 males, six females; mean age: 22.4 ± 1.6 years) or the sham group (*n* = 16; nine males, seven females; mean age: 21.9 ± 1.7 years). All participants met the same inclusion criteria as in Experiment 1 and had no history of neurological or musculoskeletal disorders. Participants were excluded if they had any severe psychiatric or other medical conditions that could interfere with the study. Among the 32 participants, six had also participated in Experiment 1. All participants received a comprehensive explanation of the experimental procedures, and written informed consent was obtained before participation. The present study was conducted in accordance with the principles outlined in the Declaration of Helsinki and was approved by the Ethics Committee of Niigata University of Health and Welfare (approval number: 19381–240809).

#### 2.3.2 tDCS

**FIGURE 2:**
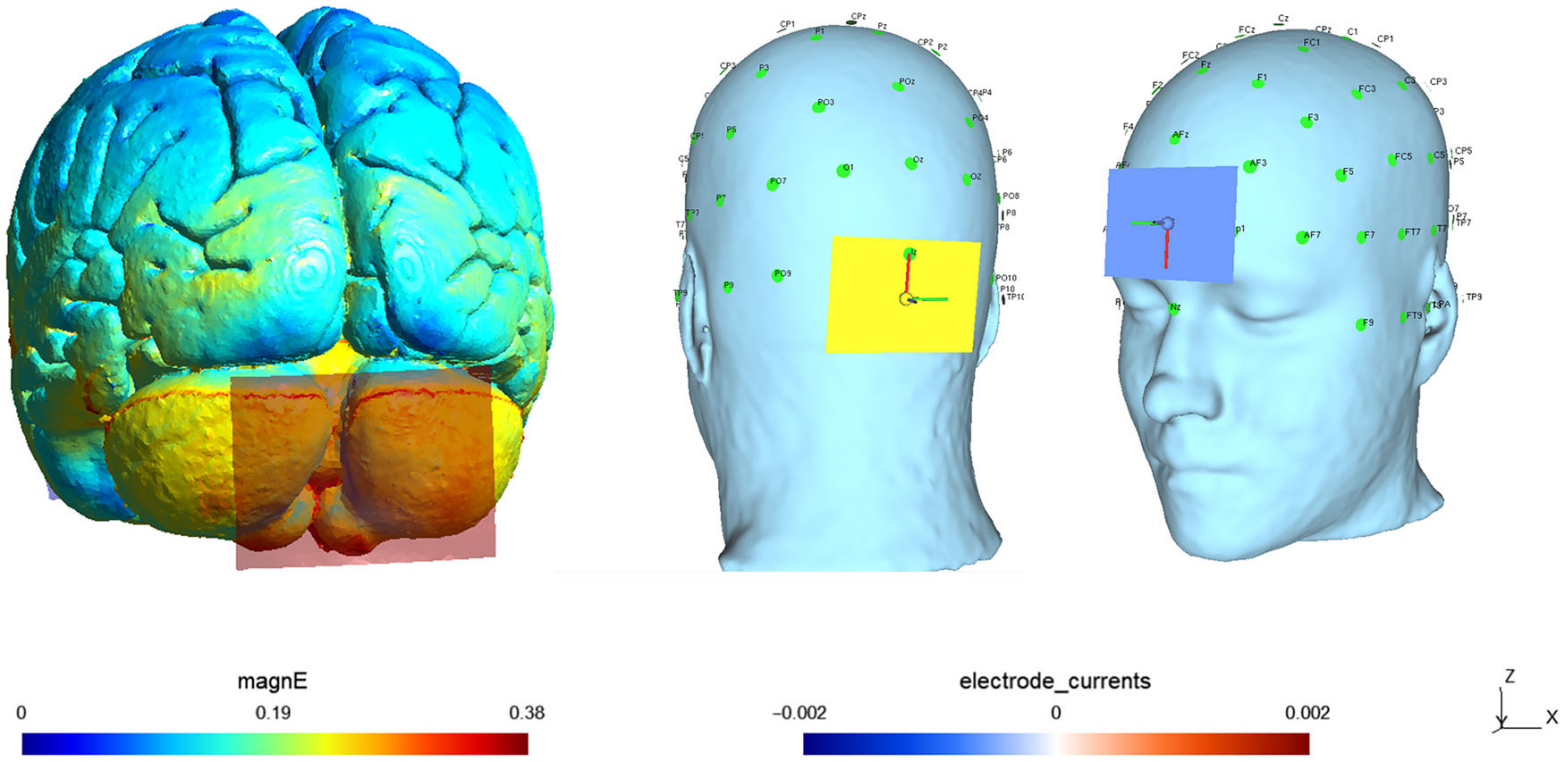
Electrode montage and SimNIBS version 4.0.1 electric field simulations used in Experiment 2.

tDCS was administered using a DC‐STIMULATOR PLUS device (Eldith, NeuroConn GmbH, Germany), with participants seated in a resting chair during the intervention. Sponge electrodes (5 × 7 cm, 35 cm^2^; current density, 0.057 mA/cm^2^) soaked in saline were used for stimulation. The anode electrode was positioned 2 cm below the external occipital protuberance, while the cathode electrode was placed at the center of the forehead. The stimulation intensity was set to 2.0 mA, with a duration of 20 min for the tDCS group and 30 s for the sham group. The fade‐in/fade‐out time was 5 s. These stimulation parameters were determined based on a previous study (Inukai et al. 2016). Electric field simulation was conducted using SimNIBS version 4.0.1 (Figure 2).

#### 2.3.3 OLS Balance Test With Eyes Closed

The OLS balance test with eyes closed was conducted as in Experiment 1, measuring the duration participants could maintain OLS on both the left and right sides. The upper limit of measurement time was 120 s, and the criteria for concluding the measurement were consistent with those used in Experiment 1 (Morioka et al. 2012). OLS time with eyes closed was measured once for each leg, and the mean of the two measurements was calculated.

#### 2.3.4 Experimental Procedures

**FIGURE 3:**
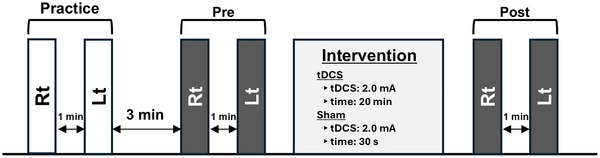
Experiment 2. OLS balance tests with eyes closed are conducted once on each side, both pre‐ and postintervention (tDCS or Sham). OLS, one‐legged standing; tDCS, transcranial direct current stimulation.

The protocol for Experiment 2 is shown in Figure 3. First, participants practiced the OLS test with eyes closed on both the left and right sides, performing the task once on each side. Preintervention, the duration of OLS with eyes closed was measured for the right leg, followed by a 1‐min rest. The same measurement was then performed for the left leg. Postintervention, the duration of OLS with eyes closed was reassessed for both the right and left legs.

#### 2.3.5 Statistical Analysis

A two‐way mixed‐design analysis of variance, including the group (tDCS, sham) as a between‐subject factor and time (pre, post) as a within‐subject factor, was used to examine the effect of tDCS on OLS balance with eyes closed. A post hoc analysis was conducted using a paired *t*‐test. All statistical analyses were performed using IBM Statistical Package for the Social Sciences Version 25 (IBM Corp., Armonk, NY, USA). Statistical significance was set to *p* < 0.05. Data were visualized through Raincloud plots generated in JASP (version 0.19.3), an open‐source statistical software program.

## 3 Results

### 3.1 Experiment 1

#### 3.1.1 OLS Time With Eyes Closed

Across all participants, the mean OLS time with eyes closed was 31.97 ± 26.32 s (mean ± standard deviation), ranging from 4.96 to 106.44 s.

#### 3.1.2 Brain Regions Associated With OLS Balance With Eyes Closed

**FIGURE 4:**
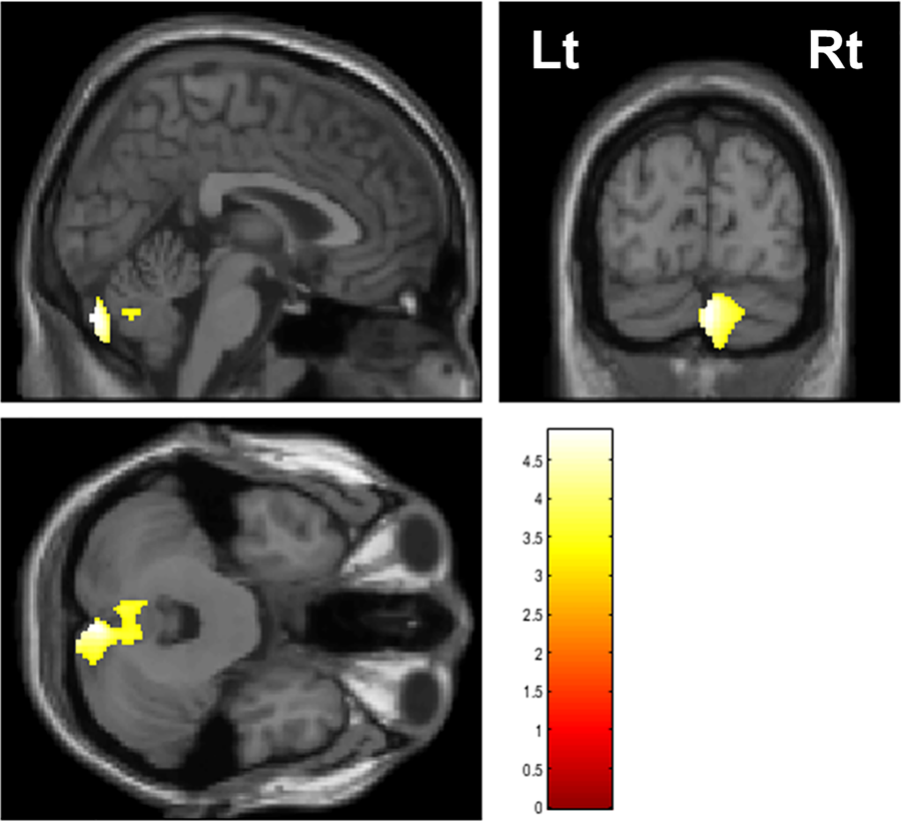
GM regions showing a significant positive correlation with OLS time with eyes closed. Individuals with shorter OLS time with eyes closed exhibit smaller GM volume in the cerebellar posterior vermis (*p* = 0.006, FWE‐corrected at the cluster level, MNI coordinates of peak voxel: *x* = 9 mm, *y* = −87 mm, *z* = −45 mm). OLS, one‐legged standing FWE, family‐wise error; GM, gray matter.

VBM analysis revealed positive correlations between OLS time with eyes closed and GM volume in the cerebellar posterior vermis (FWE cluster‐corrected *p* value = 0.006; cluster size = 2831 voxels; *T* value = 4.32; MNI coordinates of the peak voxel: *x* = 9, *y* = −87, *z* = −45; Figure 4). No brain regions exhibited a negative correlation with OLS time with eyes closed.

### 3.2 Experiment 2

**FIGURE 5:**
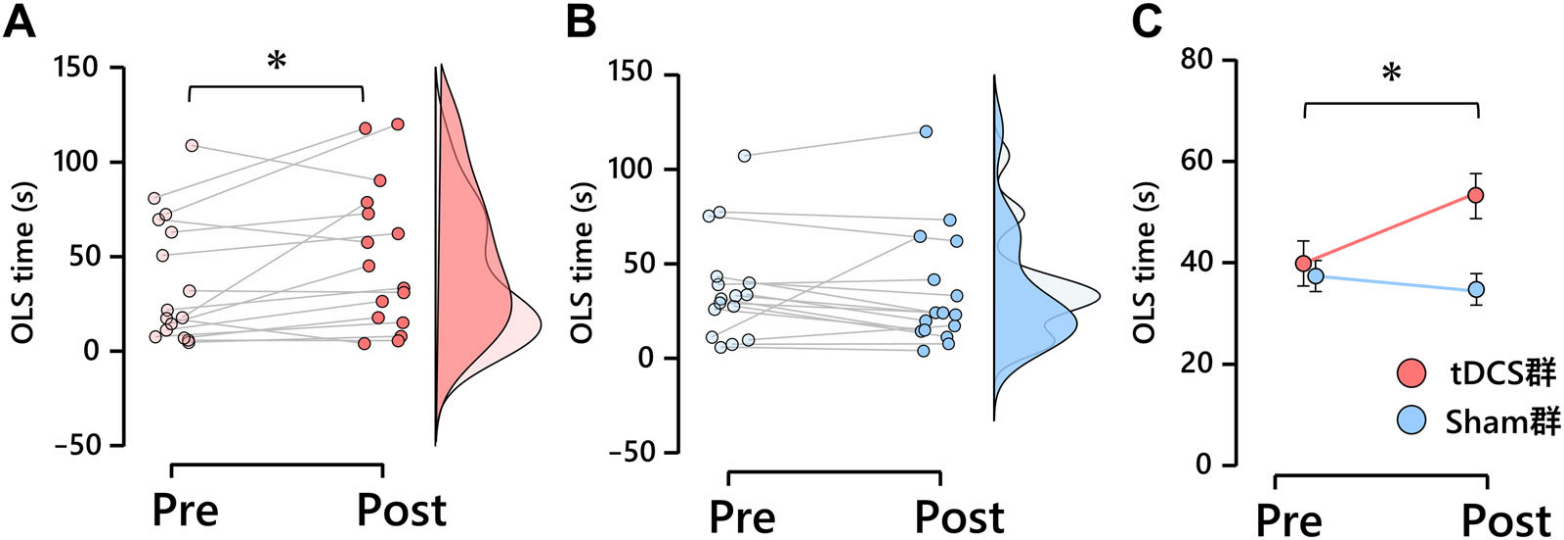
Effects of tDCS on the cerebellar vermis on OLS time with eyes closed. (A) tDCS group. (B) Sham group. The figure illustrates both individual data points with connecting lines (dots and lines, left) and one‐sided violin plots representing data distribution (right). (C) Group means are shown as circles, with error bars representing the standard error. *p < 0.05. OLS, one‐legged standing; tDCS, transcranial direct current stimulation.

Table 1 shows the mean OLS time with eyes closed for the tDCS and sham groups, along with the results of a two‐way mixed‐design ANOVA. The analysis revealed a significant interaction between time and group (*F* value (1, 30) = 4.693, *p* = 0.038). Post hoc analysis indicated that the OLS time with eyes closed was significantly better postintervention than that preintervention in the tDCS group (*p* = 0.037; Figure 5). In contrast, no significant differences were observed in the sham group (*p* = 0.555).

**TABLE 1:** OLS time with eyes closed (mean ± standard error) pre‐ and postintervention and results of two‐way mixed‐design ANOVA.

	Pre	Post	*p*‐value
tDCS group (s)	36.50 ± 8.21	49.05 ± 9.59	0.037
Sham group (s)	37.36 ± 6.96	34.72 ± 7.73	0.555

	Time	Group	Time × group
*F*‐value	2.000 (1, 30)	0.374 (1, 30)	4.693 (1, 30)
*p*‐value	0.168	0.545	0.038

Abbreviations: OLS, one‐legged standing; tDCS, transcranial direct current stimulation.

## 4 Discussion

The present study investigated whether anodal tDCS targeting brain regions associated with OLS balance with eyes closed would enhance balance performance. In Experiment 1, we used VBM to study the cortical signature related to OLS balance with eyes closed. The individuals with poor balance during OLS with eyes closed had smaller GM volume in the cerebellar posterior vermis. In Experiment 2, we investigated the effects of anodal tDCS applied to the cerebellar vermis, including posterior vermis, on OLS balance with eyes closed. The results suggest that anodal tDCS targeting this region enhances balance function.

This study suggests that GM volume in the cerebellar posterior vermis is associated with OLS balance with eyes closed in young healthy individuals. Although previous ROI‐based studies have demonstrated that the cerebellum, including the vermis, is involved in balance function, this is the first study to demonstrate, using whole‐brain VBM, a specific association between the cerebellar vermis and interindividual differences in OLS balance with eyes closed in young healthy individuals. Sensory information from vestibular, visual, and proprioceptive sensations is used in human balance control (Henry and Baudry 2019; Peterka 2002). In particular, balance control with the eyes closed relies more on proprioception, as visual input is removed (Goble et al. 2019). The cerebellar posterior vermis and the paravermal region of the cerebellum, which are identified to be associated with OLS balance with eyes closed in the present study, receive proprioceptive inputs via the spinocerebellar tract (Bosco and Poppele 2001; Jiang et al. 2015; Pop et al. 2022). Previous studies have also reported that lesions in the cerebellar posterior vermis result in a decline in standing balance function with eyes closed (Schoch et al. 2010). Furthermore, individuals with chronic ankle instability rely more on vision than the average person does and exhibit impaired OLS balance with eyes closed. GM volume in the cerebellar vermis is reduced in these individuals (Xue et al. 2021). These findings suggest that the cerebellar vermis is a brain region associated with balance control and relies on proprioception, such as in OLS balance with eyes closed. Therefore, in the present study, it is conceivable that individuals with diminished OLS balance with eyes closed may exhibit a small GM volume in the cerebellar vermis.

The results of Experiment 2 revealed that anodal tDCS targeting the cerebellar vermis, including the posterior vermis, enhanced OLS balance with eyes closed. Older individuals with impaired standing balance have declined GM volume in the cerebellar vermis (Kannan et al. 2022), and anodal tDCS applied to the cerebellar vermis improves standing balance function in this age group (Ehsani et al. 2017; Yosephi et al. 2018). The GM volume of the cerebellar vermis is decreased in patients with multiple sclerosis with proprioception and balance dysfunction (Prosperini et al. 2013). Similar to findings in older adults, anodal tDCS of the cerebellum has been reported to improve closed‐eyes balance function in patients with multiple sclerosis (Raeisi et al. 2021). Conversely, studies in young healthy adults reported that anodal tDCS applied to the cerebellum did not enhance balance performance (Foerster et al. 2017). Nevertheless, stimulation targeted the cerebellar hemispheres, and balance was assessed under eyes‐open conditions, both of which differ from the present study; these differences in stimulation site and assessment conditions may account for the discrepant findings. Here, Experiment 1 demonstrated that OLS balance with eyes closed was associated with GM volume of the cerebellar vermis, and anodal tDCS then targeted this region. Therefore, for immediate improvement in OLS balance with eyes closed in young healthy individuals, targeting the cerebellar vermis rather than the cerebellar hemispheres may be more effective.

The neurophysiological mechanisms by which anodal tDCS targeting the cerebellar vermis, particularly the posterior cerebellar vermis, enhances OLS balance with eyes closed remain unclear, but may involve inducing plastic changes in glial cells and dendritic structure. Anodal tDCS generally depolarizes the resting membrane potential of the cells directly under stimulation (Nitsche and Paulus 2000; Nitsche et al. 2008; Bhattacharya et al. 2022). This change may also influence the membrane potential of glial cells (Ruohonen and Karhu 2012; Monai et al. 2016), potentially contributing to performance enhancement. Such functional plasticity may eventually lead to structural plasticity at the dendritic level (Barbati et al. 2020; Paciello et al. 2018). Barbati et al. reported that anodal tDCS applied to M1 of mice for 20 min over 3 days resulted in increased dendritic density (Barbati et al. 2020). Furthermore, tDCS administered to the left dorsolateral prefrontal cortex for 12 days increased GM volume within the targeted region in patients with depression (Jog et al. 2023). In addition, single sessions of NIBS, such as continuous theta‐burst stimulation, have been reported to induce alterations in GM volume at the stimulation site, indicating that even short‐duration NIBS can elicit structural plasticity (Jung and Lambon Ralph 2021). Although the present study does not clarify whether structural plasticity occurred, given the short stimulation duration of 20 min, the observed improvement in OLS balance performance with eyes closed may be attributable to plastic changes in the dendrites and glial cells of cerebellar posterior vermis, which play a crucial role in this balance function.

This study demonstrated that GM volume in the cerebellar posterior vermis was associated with OLS balance with eyes closed, and that anodal tDCS applied to the cerebellar vermis, including the posterior vermis, enhanced OLS balance with eyes closed. Nonetheless, some limitations should be considered. Although anodal tDCS produced immediate improvements in OLS balance with eyes closed, it remains unclear whether these effects reflect improved balance function per se or facilitated motor learning. The cerebellum contributes to both balance control and motor learning (Ito 2000; Bernard and Seidler 2013); thus, vermis‐targeted tDCS may partly enhance motor learning rather than solely improving balance function. Additionally, this study evaluated only the immediate effects after a single tDCS session, and effect persistence was not assessed. Further work should disentangle balance function improvement from motor learning facilitation and evaluate the durability of effects over time. Moreover, consistent with prior studies, the reference electrode was placed over the frontal region. Because frontal cortical areas have been implicated in balance control, a contribution from current flow originating at the reference electrode to the observed improvements in OLS balance with eyes closed cannot be excluded. Studies using alternative reference electrode placements are warranted to better isolate cerebellar stimulation effects. Additionally, participants were limited to young healthy adults, and the sample size in Experiment 2 was relatively small. Balance performance was assessed using a single behavioral measure, namely OLS duration with eyes closed. Therefore, it remains unclear whether comparable findings would be observed in older adults or individuals with chronic ankle instability, or whether similar patterns would emerge with other balance indices, such as center of pressure measurements. Finally, the neurophysiological mechanisms underlying anodal cerebellar tDCS effects remain unclear. In Experiment 2, MRI data were not acquired from all participants; thus, the relationship between cerebellar vermis GM volume and individual responsiveness to cerebellar tDCS could not be examined. It also remains unknown whether improvements in OLS balance with eyes closed are accompanied by structural changes in cerebellar vermis GM volume. Future studies should examine associations between cerebellar GM volume and stimulation effects and longitudinally evaluate cerebellar tDCS effects on balance performance and cerebellar vermis structural plasticity.

## 5 Conclusion

The GM volume of the cerebellar posterior vermis is associated with OLS balance with eyes closed. Furthermore, anodal tDCS targeting the cerebellar vermis, including the posterior vermis, enhances balance performance during this task in healthy individuals.

## Author Contributions

Conceptualization: **Hirona Takahashi**, **Yasuto Inukai**, **Naofumi Otsuru**, and **Hideaki Onishi**. Methodology: Hirona Takahashi, **Noriko Sakurai**, and **Naoki Kodama**. Formal analysis and investigation: Hirona Takahashi, **Kazuaki Nagasaka**, and **Shota Miyaguchi**. Writing – original draft preparation: Hirona Takahashi. Writing – review and editing: Yasuto Inukai and Hideaki Onishi. Funding acquisition: Hirona Takahashi. Supervision: Hideaki Onishi.

## Funding

This work was supported by the Scientific Research of Graduate Students of Niigata University of Health and Welfare, 2024, and by JST SPRING, Grant Number JPMJSP2173.

## Ethics Statement

The present study was conducted in accordance with the principles outlined in the Declaration of Helsinki and was approved by the Ethics Committee of Niigata University of Health and Welfare (approval number: 19252–240308, 19381–240809).

## Consent

All participants were fully informed about the nature of the study, and verbal consent was obtained before their participation. The funder had no role in the study design; collection, analysis, or interpretation of the data; writing of the report; or decision to submit the article for publication.

## Conflicts of Interest

The authors declare no conflicts of interest.

## Data Availability

Data and code used in this study will be made available upon reasonable request to the corresponding author.
